# High male proportions of Nile tilapia in a zero-water exchange biofloc system even at lower methyltestosterone concentration in the feed and water temperature below ideal

**DOI:** 10.1590/1984-3143-AR2025-0118

**Published:** 2026-01-26

**Authors:** José Fernando Paz Ramírez, Érika Ramos de Alvarenga, Franklin Fernando Batista da Costa, Kelly Moura Keller, Ana Paula Campos, Natan Paulo Bento Pio, Rafael Hiroaki Ito, Lee Deyver Carvalho Pena Mansur, William Gleidson Alves Torres, Vinícius Monteiro Bezerra, Dara Cristina Pires, Laryssa Evelyn Santos Soares, Guilherme Figueira Gonçalves, Gabriela Lago Biscoto, Marcelo Rezende Luz, Eduardo Maldonado Turra

**Affiliations:** 1 Escola de Veterinária, Universidade Federal de Minas Gerais, Belo Horizonte, MG, Brasil; 2 Empresa de Pesquisa em Agropecuária de Minas Gerais, Felixlândia, MG, Brasil

**Keywords:** NGTAqua Chitralada lineage, bioflocs, methyltestosterone residues, temperature, environmental sustainability

## Abstract

Since temperature plays an important role in feed consumption, chemical reactions and metabolism, it was hypothesized that it is below ideal it could interfere in the masculinization of tilapia in biofloc system (BFT) and the ideal methyltestosterone (MT) concentration in the feed. The masculinization rates of Nile tilapia in a zero-water exchange BFT (and without clarification) at 25°C and 28 °C combined with concentrations of MT in the feed (0 (control), 10, 20, 30 and 40 mg ∙ Kg^-1^ of feed) were evaluated using 3 replicates per treatment (30 tanks of 50 liters, 2 larvae ∙ L^-1^). Larvae were fed five times a day for 28 days. The water quality and growth performance did not diverge between MT concentrations (p > 0.05). Larvae grew 2.7 times higher in 28°C than 25°C. The control treatments did not differ from each other for male proportion (mean = 66,75%) but differed from all hormonal treatments. These treatments presented masculinization rates above 98.7% and 96.9%, at temperatures of 25°C and 28°C, respectively, and did not differ from each other at the same MT concentrations. Therefore, it is feasible to use an even lower concentration (10 mg of MT ⋅ Kg^-1^ of feed) in a zero-water exchange BFT, regardless of these temperatures. At the lower temperature, the input of MT in the system was smaller due to smaller feed intake, however the fingerlings would take longer to reach commercial body weight. Furthermore, after 2 hours of the last hormone feeding, MT residues were not detected in any biofloc/water mixture samples.

## Introduction

For tilapia grow-out, sexual precocity is not interesting, as it generates heterogeneous batches, loss of population and production control, inadequate feeding strategy, reduced growth (Farahmand et al., 2007; Baroiller et al., 2014), as well as excess management and reduction water quality. The production of monosex tilapia is a form of control of these negative impacts of reproduction. There are various techniques for reaching populations of only one sex, and one of these uses male and female steroid sex hormones offered in the diet (Karsli, 2021). In the case of tilapia, the choice is to produce male individuals, obtained using male hormones, a technique most applied due to its easy application, low cost and greater effectiveness (Baroiller and D'Cotta, 2018; Costa e Silva et al., 2022). The hormone most used in this technique is 17α-methyltestosterone (MT), due to its high capability of masculinization (Sarker et al., 2022).

Several studies have proven that the use of MT in tilapia production does not contaminate the meat of the fish produced (Rothbard et al., 1990; Curtis et al., 1991; Piferrer, 2001) and does not pose a risk to human health (Baroiller and D 'Cotta, 2018; Thanasupsin et al., 2021). However, this procedure has environmental impacts as it is generally carried out in hatcheries, where the exchange of water and disposal of effluents can contaminate natural bodies of water (Baroiller and D'Cotta, 2018; Ramírez et al., 2024). MT degradation and biotransformation pathways, which occur in the environment and in fish, have not yet been completely elucidated (Lone and Ridha, 1993; Mlalila et al., 2015). Therefore, it is not yet known whether the metabolites formed after the metabolization of MT continue to have any potential for steroid action.

In a recent study, Ramírez et al. (2024), using a water temperature of 28°C, managed to reduce the use of MT in the masculinization of tilapia in a zero-water exchange BFT system to 30 mg ∙ Kg^-1^ of feed, half of what is commonly used in earthen ponds (Baroiller and D’Cotta, 2018), reaching 99% of males. These authors did not detect MT residues in the water 12 hours after the last hormone-containing feeding. As a result, the environmental problem related to the use of MT in tilapia masculinization was minimized using a BFT system with zero-water exchange, thus paving new paths towards a sustainable tilapia hatchery. Temperature directly affects the environment and the metabolic rates of aquatic organisms (Rubalcaba et al., 2020), that is, it interferes both with water parameters and with the well-being, consumption, and growth performance of fish. Among the abiotic factors that can interfere with gonadal differentiation, temperature is the one that stands out the most (Baroiller and D’ Cotta, 2001; Yao et al., 2021). The explanation for sexual inversion due to temperature in fish lies in the ability of the enzyme cytochrome P450-aromatase to catalyze the conversion of testosterone into 17β-estradiol, a hormone that acts in the differentiation and development of the ovaries. Heat treatment has a negative regulatory effect on the expression of this enzyme (Nakamura et al., 2015; Wan et al., 2016; Pandit et al., 2015; Nozu and Nakamura, 2020). Thereby, in tilapia larvae subjected to higher temperatures during the differentiation period, masculinization of XX females occurs due to the blockage of this enzyme (Guiguen et al., 1999; Baroiller and D’Cotta, 2001). Recently, the use of heat treatment for masculinization has been gaining prominence, however, the temperatures used in this technique are generally well above the range between 27° and 30°C, considered ideal for Nivelle et al. (2019), resulting in high mortalities (Borges et al., 2005), and raising questions related to animal welfare. Outside the temperature range considered optimal, fish can experience a stressful situation, leading to physiological dysfunctions that can impair performance and even lead to death (Pörtner et al., 2017; Yang et al., 2021; Bezerra et al., 2025).

At temperatures above the ideal limit, a reduction in fish consumption and growth performance may occur (Islam et al., 2020; Khieokhajonkhet et al., 2022). A study carried out by Zhou et al. (2022), exposed tilapia to a temperature of 36°C, and observed that there was an increase in ventilatory frequency, a decrease in tolerance to hypoxia and a 21% increase in mortality when compared to tilapia kept in water at 28°C. Borges et al. (2005), comparing the masculinization rates of Nile tilapia of the Chitralada lineage at temperatures 27°C and 35°C, achieved rates of approximately 62% and 72%, respectively. Nevertheless, they attributed it to the higher temperature and greater mortality caused by cannibalism. However, higher temperatures within the optimal range for each species can stimulate feed intake, enhance digestion, and improve growth performance (Neuheimer et al., 2011; Volkoff and Rønnestad, 2020). They may also promote higher masculinization rates in Nile tilapia larvae, even when hormone concentrations in the feed are reduced (Ramirez et al., 2024), particularly in more thermosensitive lineages (Baroiller and D’Cotta, 2018).

Therefore, it is interesting to understand whether a temperature within the limits of thermal confort (28°C) and a lowest one below this limit (25°C) promotes in tilapia masculinization rates in the BFT system with zero-water exchange and without clarification, especially when combined with lower MT concentrations. At 25°C, larval metabolic activity is likely to decrease, leading to reduced feed and hormone intake. This reduction could negatively affect larval masculinization, potentially requiring a different minimum hormone concentration in the diet compared to 28°C. A temperature of 25°C is also commonly observed throughout the year in Nile tilapia hatcheries in tropical regions, even when tanks are installed under greenhouses. Therefore, it is important to assess Nile tilapia masculinization in biofloc systems with zero water exchange under such suboptimal conditions.

To reduce the environmental impact and find the lowest masculinizing concentration possible, this study evaluated the masculinization rate of Nile tilapia in the biofloc system (BFT) with zero water exchange in temperatures of 28°C and 25°C. For this experiment, MT concentrations from 0 to 40 mg ∙ Kg^-1^ of feed were tested, with two concentrations (10 and 20 mg ∙ Kg^-1^ of feed) below the lowest masculinization rate (30 mg ∙ Kg^-1^ of feed and 99% masculinization rate) obtained by Ramírez et al. (2024) in a study with masculinization of tilapia in the same system. Furthermore, it was studied how these temperatures could interfere with water quality, growth performance, the masculinization proportion, the amount of hormone used and the MT residues on effluents.

## Methods

### Experimental design

The experiment was carried out at the Aquaculture Laboratory (LAQUA) of the Veterinary School of the Federal University of Minas Gerais (UFMG). All procedures were previously reviewed and approved by the Ethical Committee on Animal Use of UFMG (CEUA/UFMG) under protocol number 66/2023.

For the experiment, Nile tilapia larvae (11. 57 ± 0.46 mg and 0.93 ± 0.03 cm) were used from spawning and hatching, on the same day, of 12 females (average weight of 766.7 ± 97 g) from the ninth generation of the genetic improvement program for body weight increase (more details are available in Cavatti Neto et al., 2023) from the NGT-Aqua (Nutrition, Genetics and Technology for Aquaculture) research group, belonging to the Aquaculture Laboratory/ UFMG.

Soon after yolk sac absorption, the larvae were transferred to 30 polyethylene tanks (50 L useful volume). The tanks were filled with pre-matured biofloc previously developed in other tanks where adult fish have been stocked (25 kg of adult fish ∙ m^-3^) through the entire year, by supplementing with cane sugar as the carbon source to maintain a carbon: nitrogen ratio close to 6: 1, important for heterotrophic bacteria to develop and remove ammonia from the water, together with nitrifiers bacteria (total ammonia nitrogen 0.06 mg ∙ L^-1^ ± 0.03 mg ∙ L^-1^; nitrite 0.85 mg ∙ L^-1^ ± 0.08 mg ∙ L^-1^ and nitrate 71.93 mg ∙ L^-1^ ± 14.07 mg ∙ L^-1^). A total of 3,600 larvae were distributed in the tanks (120 larvae ∙ tank^-1^) and it was ensured that each tank of each treatment received the same number of larvae from each of the 12 females. The experimental design was completely randomized, with treatments resulting from the combination of 2 cultivation temperatures (25°C and 28°C) and five levels of methyltestosterone (MT) in the diet (0, 10, 20, 30 and 40 mg ∙ Kg^-1^ of feed) for 28 days. Shorter treatment period than 28 days was not tested because according to previous study of our research group (Costa et al., 2024), the masculinization proportion for this Chitralada lineage in zero exchange water BFT up to 21-days MT treatment period in 28.4°C ± 1.4 was 87.68% (from 80.94 to 93.60%), a male proportion not high enough to be used for commercial purpose.

An initial biometry was carried out with 20 animals, randomly removed from each experimental unit, establishing the initial stocking density of 2 larvae ∙ L^-1^ (n = 100 larvae ∙ tank^-1^). The 20 larvae were weighed together on an analytical balance (Marte Científica, Brazil), with a precision of 0.001 g. Then, the larvae were euthanized with eugenol (180 mg ∙ L^-1^ for 10 min.) to have their body length measured.

The Nile tilapia larvae were fed with a commercial ration (Propescado-Nutriave Foods) containing 55% crude protein, 12% moisture, 10% ether extract and 15% ashes. Each experimental diet received the masculinizing hormone using 99.5% P.A ethyl alcohol as a vehicle for 17α-methyltestosterone. The solution was previously prepared by weighing the hormone and diluting it in 200 mL of ethyl alcohol. After preparing the solution, the liquid was distributed over the diet using a 500 mL spray bottle. Then, the diet was stored away from light to evaporate the alcohol. The diets were identified and stored in a freezer at -20 °C, protected from light, to ensure MT stability throughout the experiment.

The larvae were fed five times a day at a feeding rate of 30%, 25%, 20% and 15% of body weight in the first, second, third and fourth week, respectively, according to Costa e Silva et al. (2022) and Ramirez et al. (2024). Feeding correction was guided by weekly biometrics, carried out with samples from 20 larvae per tank, estimating a probable weight gain in the following week based on the weight gain of the previous week and a mortality of 3% for each week.

Treatments at 28°C had a higher feed consumption than treatments at 25°C, therefore, the amount of hormone per larvae was different between these two groups, as calculated below. Considering the treatment of 40 mg of MT ∙ Kg^-1^ of feed, as a reference, at 25°C treatments, the amounts of feed and hormone offered to each larvae during the experimental period were, respectively, 236.14 mg of feed and 9.45 µg of MT for each fish, at 25°C temperature. In contrast, at 28° C, 775.72 mg of feed were used and 31.03 µg of MT for each fish at 28°C treatments.

In the period after masculinization, the fish received a hormone-free diet until they reached an adequate size for sexing (>3.2 cm) using the aceto-carmine technique (Guerrero and Shelton, 1974).

### Water quality

To maintain constant experimental temperatures, the tanks were installed in agricultural greenhouses, and each one received 100 Watt thermostats. Aeration was maintained by radial blowers connected to microporous hoses installed in each tank to maintain adequate oxygen levels for the species. In addition, during the experiment, 75 g of limestone powder were added in each tank, as a carbonates source, to maintain alkalinity at levels suitable for nitrifying bacteria and the pH close to neutrality.

During the entire experimental period, there was no water renewal or solids removal from the tanks (i.e. without clarification), with the volume of water lost through evaporation being supplemented, daily, with clear water from an artesian well.

Temperature, pH, oxygen, and salinity measurements were taken three times a week in the tanks of all treatments. To monitor and ensure that thermal parameters were maintained at 25°C and 28°C according to the treatment, Exbom® digital thermo-hygrometers were used, where the minimum and maximum temperatures were evaluated daily. With these measures, thermostats could be adjusted to the desired temperature. The nitrogenous compounds total ammonia nitrogen (TAN), non-ionized ammonia (NH_3_) and nitrite (NO_2_^-^) were measured twice a week. Analyzes of alkalinity and settleable solids (SS) were carried out once a week, and nitrate (NO_3_^-^), total nitrogen (N), total suspended solids (TSS) once, at the end of the experiment.

Dissolved oxygen levels (mg ∙ L^-1^) were measured using an AT 155 oximeter (Alfakit®, Florianópolis, Santa Catarina, Brazil); and pH, salinity (g ∙ L^-1^) and temperature (ºC) were monitored using a multiparameter probe (Hanna®, Barueri, São Paulo, Brazil). The levels of total ammonia nitrogen (TAN) and nitrite (NO_2_^-^) were measured according to the methodologies established by UNESCO (1983) and Bendschneider and Robinson (1952), respectively, while the nitrate (NO_3_) was quantified using the method described by Monteiro et al. (2003).

Alkalinity was measured using the methodologies proposed by APHA (1998). Settleable solids (SS) were quantified using Imhoff cones (Avnimelech, 2006). Total suspended solids (TSS) were measured after collecting 20 mL of water, with subsequent filtration through GF50-A glass fiber filters, which were then dried and weighed to quantify the retained material.

### Growth performance, survival, and masculinization rate

The growth performance of tilapia was evaluated by the final body weight (BWf), final body length (BLf), specific growth rate (SGR), Fulton condition factor (CF) and survival (S), at the 29^th^ day, in the morning, before the first feeding of the day, as follows: final body weight (BWf) = mean of the mass (g) of 20 larvae randomly picked per experimental unit; final body length (BLf) = mean of body length (cm) of 20 larvae randomly picked per experimental unit; specific growth rate (SGR) = 100 × (log (final body weight) – log (initial body weight)) / days of experiment; condition factor (CF) = final body weight (g) × total body length^-3^ (cm) x 100; survival (S) = (final number of individuals / initial number of individuals) x 100; feed consumption = amount of feed offered (g); biomass = BWf (g) × final number of individuals; productivity = biomass (kg) / 0.05 m^-3^ = kg×m^-3^; feed conversion ratio (C.A.) = feed consumption (g) / biomass (g).

After a period of 28 days of experiment, the fingerlings from each treatment were removed, as they reached the minimum size of 3.5 cm for sexing, to verify the effect of MT concentrations on masculinization. For this analysis, tilapias were euthanized via eugenol-induced overdose (180 mg ∙ L^-1^ for 10 min.) (Vidal et al., 2008). The fingerlings were fixed in Bouin's liquid for 24 hours and preserved in 70% ethanol. Subsequently, the gonads were removed, stained with the aceto-carmine according to Guerrero and Shelton (1974) and analyzed under an optical microscope (40x magnification).

### MT residues in the water

The evaluation of MT input into the system was carried out based on the amount of feed offered and the concentration of MT used in each treatment. For each treatment, three 40.0 mL water × biofloc mixture samples were collected 2 hours after the last feeding with hormone-containing food and for the subsequent 10 hours, placed in 50 mL plastic centrifuge tubes, and stored at -20 °C in the dark until analysis. It is important to highlight that water × biofloc mixture sample contains water, flocs and suspended feces (tilapia feces has a difficult to decante) and the sample were not previously decanted and filtered (settleable and total suspended solids were mantained). MT analysis in water was first validated in-house so that water samples from the tanks could be evaluated. The chemicals and reagents used, the sample preparation, the preparation of calibration standards, the instruments and HPLC conditions and the parameters for method validation followed Ramirez et al. (2024).

### Statistical evaluation

For all variables (except for masculinization proportions), linear regression models were adjusted, considering temperature as a categorical variable (25°C = 0 (indicator variable); 28°C = 1) and the hormone concentration as a continuous variable. When the assumptions of normality and homogeneity of variances were violated based on the Shapiro-Wilk and Bartlett tests, respectively, log data transformation and/or weighted least square method were used. Differences were considered significant when *p < 0.05*. The number of males and non-males of each treatment was determined and the masculinization proportions were analyzed by Chi-square test of independence to determine whether there is an association between categorical variables, i.e., whether the masculinization proportions are independent or related to experimental groups (Conover, 1999). Then, the association test of the male proportions between pairs of treatment were performed with Fisher Exact with Bonferroni correction. Since significance values have been adjusted by Bonferroni correction for multiple tests, the p-value was 0.001 (0.05/45) for forty-five pairwise comparisons in this statistical analysis. Statistical analyzes were performed using the InfoStat program (Di Rienzo et al., 2015) and R software (R Core Team, 2021).

## Results

### Water quality

All water parameters were within the reference values ​​for the species, except for the temperature, which was below the recommended level due to the tested factor itself, and the settleable solids, which were below ideal due to small biomass of larvae, that result in small imput of feed and nitrogen residues in the water and, consequently, few nutrients for heterotrophic bacteria growth, The salinity of this experiment showed values between 0.12 and 0.14 g ∙ L^-1^, that are close to 0.00 g ∙ L^-1^, ideal for masculinization in BFT (Do Valle et al., 2023).

The average temperature values ​​were consistent with the treatments applied. Throughout the experimental period, the 25°C treatment had a mean of 24.79°C and there was a mean significant difference of +3.73°C (*p < 0.0001*) between this value and the average of the ​​28°C treatment (Table 1), regardless the MT concentration (non-significant interaction), such as it was expected due to temperature control with heaters thermostats to maintain the two different temperatures defined by the experimental design.

**Table 1:** Means ± standard deviation of water quality variables per temperature (T, 25°C or 28°C), in combination with four different concentrations of 17α-methyltestosterone (MT) in the diet (0, 10, 20, 30 and 40 mg ∙ Kg^-1^ of feed) and p values associated with sources of variation and their interaction (T × MT) from Nile tilapia masculinization in a biofloc system.

Variables	T	MT					p-values			Regression models; adjusted R^2^	1Reference values
		0	10	20	30	40	T	MT	T*MT		
Temperature (°C)	25°C	24.76 ± 0.06	24.68± 0.12	24.92± 0.15	25.30± 0.22	24.70± 0.24	<.0.0001	0.408	1.0	Y= 24.79 + 3.73×T; adjR^2^= 0.98	27-32a
	28°C	28.34 ± 0.14	28.62± 0.34	28.70± 0.23	28.77± 0.18	28.56± 0.50					
pH	25°C	6.99 ± 0.08	6.98± 0.03	6.94± 0.05	7.01± 0.02	7.01± 0.09	0.0032	0.5245	0.6033	Y= 6.97 - .14×T; adjR^2^= 0.57	6-9b
	28°C	6.87 ± 0.09	6.77± 0.01	6.86± 0.10	6.85± 0.06	6.83± 0.06					
Oxygen (mg.L^-1^)	25°C	6.99 ± 0.15	6.98± 0.14	6.89± 0.05	6.87± 0.20	7.01± 0.16	0.0372	0.7721	0.6318	Y= 6.96 + .22×T; adjR^2^= 0.22	>4^b^
	28°C	7.14 ± 0.10	7.23± 0.20	7.22± 0.23	7.00± 0.03	7.10± 0.21					
Settleable solids (mL∙L^-1^)	25°C	.93 ± 0.20	1.02± 0.65	1.39± 0.65	2.39± 0.38	2.02± 0.77	<.0.0001	0.084	0.3819	Y= .84 + 5.65×T; adjR^2^= 0.90	25-50c
	28°C	6.14 ± 0.25	7.12± 0.40	8.42± 0.70	7.91±1.70	8.99±3.55					
Total suspended solids (mg∙L^-1^)	25°C	1.50 ± 0.61	0.77± 0.55	4.33±1.80	4.67±2.76	1.80± 0.40	<.0.001	0.6167	0.2536	Y= 1.71 + 13.52×T; adjR^2^= 0.54	<1000d
	28°C	16.0 ±4.85	12.59±9.89	14.10±11.36	12.23±2.04	11.09±4.43					
Alkalinity (mg de CaCO_3_∙L^-1^)	25°C	92.33 ±3.06	84.0±8.72	75.67±12.42	95.67±11.85	93.0±6.93	0.0026	0.4974	0.4079	Y= 85.53 – 21.6×T; adjR^2^= 0.43	>20^b^
	28°C	70.0 ±7.94	56.33±4.16	76.33±11.93	73.0±6.08	79.33±4.04					
TAN (mg∙L^-1^)	25°C	0.06 ± 0.03	0.02± 0.01	0.01± 0.01	0.002± 0.002	0.01± 0.02	0.1711	0.0122	0.0320	Log(Y) = -3.31 – 0.08×MT + 0.09×T×MT; adjR^2^= 0.15	<1^d^
	28°C	0.02 ± 0.01	0.03± 0.04	0.005± 0.005	0.03± 0.03	0.04± 0.03					
Nitrite (NO_2_) (mg∙L^-1^)	25°C	1.10 ± 0.33	0.86± 0.29	1.24± 0.27	1.39± 0.69	1.72± 0.48	0.5513	0.0086	0.0043	Y= 0.94 + 0.017×MT - 0.026×T×MT; adjR^2^= 0.41	<8^a^
	28°C	1.12 ± 0.27	0.92± 0.28	0.84± 0.15	0.81± 0.18	0.72± 0.13					
Nitrate (NO_3_) (mg∙L^-1^)	25°C	76.23 ± 27.41	44.18±26.29	45.42±24.80	72.61±38.58	51.39±21.25	<0.001	0.8198	0.9519	Log(Y) = 3.97 + 1.38×T; adjR^2^= 0.59	<500e
	28°C	144.3±27.41	378.8±163.2	207.9±140.5	214.6±24.32	167.8±84.14					

1 Reference values: ^a^El-Sayed (2019); ^b^Wedemeyer (1996); ^c^Hargreaves (2013); ^d^Avnimelech (2009); ^e^Monsees et al. (2017). Letter “T” is an indicator variable that receives the value one (1) if the temperature treatment was 28°C and the value zero (0) if the temperature treatment was 25°C.

The pH and dissolved oxygen levels varied slightly between treatments. The 25°C treatment had a mean of 6.97 and 6.96 mg∙L^−1^, for the respective water quality variables. The 28°C treatment had a decrease of – 0.14 (*p = 0.0032*) and an increase of + 0.22 mg∙L^−1^, respectively to the two variables, regardless the MT concentration (non-significant interaction with temperature).

The settleable solids and total suspended solids had a significant increase due to the temperature. At 25°C, the mean value was 0.8 mL∙L^−1^ and 1.7 mg∙L^−1^, respectively for these water quality variables. The 28°C treatment had an increase of + 5.7 mL∙L^−1^ (*p < 0.0001*) and + 13.5 mg∙L^−1^ (*p < 0.001*), respectively to the two variables and regardless the MT concentration and the interaction with temperature. However, the mean alkalinity value decreased with increasing temperature. At 25°C, the mean value was 85.5 mg CaCO_3_∙L^−1^ and at 28°C, there was a decrease of – 21.6 mg of CaCO_3_∙L^−1^ (*p = 0.0026*), also without MT concentration effect and its interaction with temperature.

Total ammonia and nitrite showed low concentrations for all treatments. For total ammonia there was a slight reduction by the increase of MT concentration (*p = 0.0122*) and an increase by the T × MT interaction (*p = 0.0320*). At the control treatments (0 mg of MT∙Kg^−1^ of feed) the mean value was 0.03 mg∙L^−1^. At 25°C, values ​​ranged from 0.002 ± 0.002 mg∙L^−1^ (MT = 30 mg∙Kg^−1^) to 0.06 ± 0.03 mg∙L^−1^ (MT = 0 mg∙Kg^−1^). At 28°C, concentrations varied between 0.005 ± 0.005 mg∙L^−1^ (MT = 20 mg∙Kg^−1^) and 0.04 ± 0.03 mg∙L^−1^ (MT = 40 mg∙Kg^−1^). For nitrite, there was a slight increase by the increase of MT concentration (*p = 0.0086*) and a decrease by the T × MT interaction (*p = 0.0043*). At the control treatments (0 mg of MT∙Kg^−1^ of feed) the mean value was 0.94 mg∙L^−1^. At 25°C, values ​​varied between 0.86 ± 0.29 mg∙L^−1^ (MT = 10 mg∙Kg^−1^) and 1.72 ± 0.48 mg∙L^−1^ (MT = 40 mg∙ kg^−1^). At 28°C, values ​​ranged between 0.72 ± 0.13 mg∙L^−1^ (MT = 40 mg∙Kg^−1^) and 1.12 ± 0.27 mg∙L^−1^ (MT = 0 mg∙ kg^−1^).

Nitrate and total nitrogen were positively influenced by temperature (*p < 0.001*), presented mean values ​​at 25°C of 52.98 mg∙L^−1^ and 12.3 mg∙L^−1^, and at 28 °C of 210.6 mg∙L^−1^ and 47.94 mg∙L^−1^, respectively.

### Growth performance, survival and masculinization rate

The initial body weight and length of the fry did not show significant differences between all treatments, with a mean value of 11.64 mg and 0.92 cm (Table 2), showing a good initial distribution of the larvae in the tanks.

**Table 2:** Means ± standard deviation of performance variables per temperature (T, 25°C or 28°C), in combination with four different concentrations of 17α-methyltestosterone (MT) in the diet (0, 10, 20, 30 and 40 mg ∙ Kg^-1^ of feed) and p values associated with sources of variation and their interaction (T × MT) from Nile tilapia masculinization in a biofloc system.

Variables	T	MT					p-values			Regression models; adjusted R^2^
		0	10	20	30	40	T	MT	T*MT	
Initial body weight (mg)	25°C	11.91± 0.48	11.18± 0.14	11.57±0.25	11.78±0.52	11.45±0.34	0.520	0.643	0.793	Y= 11.64
	28°C	11.44± 0.27	11.53± 0.30	11.10±0.07	11.70±0.07	11.15±0.10				
Final body weight (mg)	25°C	319.6±147.1	236.5±48.01	248.8±10.45	312 ± 27.76	293.69±4.16	<.0.0001	0.891	0.129	Y= 277.4 + 475.4×T; adjR^2^= 0.90
	28°C	772.2±111.3	803.1±126.3	752.4±126.3	739.3 ± 295.6	933 ±141.5				
Initial body length (cm)	25°C	0.90± 0.05	0.95± 0.01	0.94± 0.01	0.94± 0.03	0.94± 0.04	0.713	0.269	0.297	Y= 0.92
	28°C	0.93± 0.03	0.91± 0.02	0.92± 0.05	0.95± 0.03	0.90± 0.03				
Final body length (cm)	25°C	2.39± 0.22	2.30± 0.10	2.29± 0.03	2.46± 0.05	2.37± 0.01	<.0.0001	0.714	0.338	Y= 2.34 + 0.99×T; adjR^2^= 0.91
	28°C	3.32± 0.25	3.44± 0.32	3.38± 0.22	3.33± 0.43	3.57± 0.20				
Specific growth rate (%∙day^-1^)	25°C	11.52±1.48	10.85± 0.77	10.96± 0.23	11.69± 0.45	11.59± 0.06	<.0.0001	0.566	0.855	Y= 11.12 + 3.76×T; adjR^2^= 0.80
	28°C	15.02± 0.46	15.48± 0.81	14.17±1.10	14.49±1.55	15.79± 0.55				
Condition Factor	25°C	2.23± 0.40	1.94± 0.15	2.08± 0.17	2.10± 0.06	2.22± 0.06	0.893	0.851	0.391	Y= 2.09
	28°C	2.13± 0.25	2.19± 0.19	1.85± 0.20	1.92± 0.14	2.05± 0.04				
Survival (%)	25°C	97.00±4.24	95.00±2.83	93.67±3.79	94.50±7.78	94.33±1.53	0.126	0.639	0.629	Y= 95.89
	28°C	89.67±5.69	91.00±3.00	90.67±3.06	88.33±13.20	92.00±6.08				
Productivity (Kg·m^-3^)	25°C	0.68± 0.34	0.42± 0.14	0.47± 0.03	0.55± 0.09	0.55± 0.01	<.0.0001	0.947	0.127	Y= 0.53 + 0.81×T; adjR^2^= 0.88
	28°C	1.38± 0.12	1.44± 0.26	1.37± 0.29	1.46± 0.04	1.72± 0.30				
Feed consumption (g)	25°C	41.48±1.17	45.68± 0.72	43.75± 0.74	43.69±1.06	46.11±1.12	<.0.0001	0.348	0.345	Y= 42.37 + 80.02×T; adjR^2^= 0.99
	28°C	117.9±7.8	131.5± 0.59	123.3±1.49	127.0± 0.80	129.6± 0.71				
Feed conversion ratio	25°C	1.37± 0.68	1.84± 0.03	1.88± 0.11	1.61± 0.25	1.66± 0.003	0.523	0.663	0.456	Y= 1.63
	28°C	1.72± 0.22	1.68± 0.40	1.85± 0.39	1.74± 0.03	1.54± 0.25				

Final body weight and length were higher at 28°C, presenting an average increase of + 475.4 mg and 0.99 cm (*p < 0.0001*) in relation to 25°C treatment. The mean values at 25°C were 277.4 mg and 2.34 cm (intercepts of the models fitted) and at 28 °C were 752.8 mg (significant intercept (277.4 mg) + significant temperature effect (475.4 mg) = 752.8 mg, according to the model fitted; 2.7 times larger than the body weight at 25°C) and 3.33 cm, respectively.

Following the final body weight and length, the specific growth rate of 28°C treatments were 3.8%∙day^−1^ higher than 25°C (*p < 0.0001*), presented mean value of 11.2%∙day^−1^ at 25°C and 15.0%∙day^−1^ at 28°C, regardless MT concentration effect and its interaction with temperature. However, condition factor and survival values ​​did not show significant differences between treatments, with average values of 2.1 and 95.9%, respectively (Table 2).

The increase in the final body weight at 28°C and the equivalence of survival led to a higher productivity (+ 0.8 Kg·m^-3^, *p value < 0.0001*) and higher feed consumption (+ 80.0 g, *p value < 0.0001*) at the treatments this temperature was applied. However, feed conversion ratio did not show significant differences between treatments, with an average value of 1.6 (Table 2).

**Figure 1:**
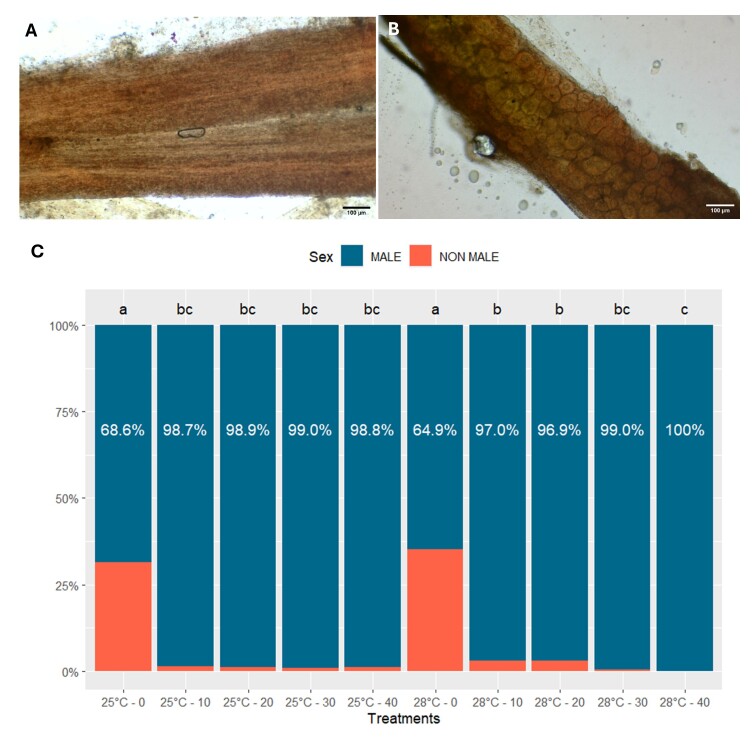
Results of sexing analysis of Nile tilapia fed diets containing different doses of 17α-methyltestosterone during the masculinization phase. A) Microscopic image of a male gonad subjected to the aceto-carmine technique from an animal in the control group of Nile tilapia produced using biofloc technology (BFT). B) Microscopic image of female gonad subjected to the aceto-carmine technique from a Nile tilapia control animal produced using biofloc technology (BFT). C) Proportion of males and non-males (females and intersex) of Nile tilapia during 28 days of masculinization under different doses of 17α-methyltestosterone in the diet (0, 10, 20, 30 and 40 mg ∙ Kg^-1^ of feed) at 25 °C and 28 °C of temperature in biofloc technology (BFT).

Significant differences were observed in male proportions, between the control and the other MT concentrations, regardless of the temperatures (*p < 0.05*) (Figure 1). The proportion of non-males (females and intersex) in the control, without the presence of hormones in the feed, was 31.4% and 35.1% at 25°C and 28°C, respectively. No differences were observed between the male proportion of MT concentration treatments ranging from 10 to 40 mg of MT ⋅ Kg^-1^ of feed, at 25°C (*p > 0.05*) and no difference between them and the respective treatments at 28°C (*p > 0.05*). However, there were differences between the proportion of males of 10 and 20 mg of MT ⋅ Kg^-1^ of feed (97% and 96.9%, respectively), and 40 mg of MT ⋅ Kg^-1^ of feed (100%) at 28°C (*p > 0.05*).

## MT residues in the water

The results of validation and the analysis of the water samples are presented below.

### Selectivity

To assess selectivity, the chromatograms of the blank sample extracts were compared with those of the fortified samples. The absence of signals, considering the respective retention times and signal-to-noise ratios, indicated that no matrix interference occurred at the same retention time as the target analyte (MT). This confirmed the excellent selectivity of the method (Ribani et al., 2004).

### Precision and accuracy

The precision and accuracy of the method were evaluated by assessing both inter- and intra-day variability for MT at four different concentration levels. The results, shown in Table 3, revealed precision values expressed as relative standard deviation (RSD) ranging from 6.28% to 8.81%. These values were consistent with the guidelines provided by INMETRO (2020), remaining within the acceptable limit of 20%. Accuracy, expressed as recovery percentage, ranged from 70.79% to 99.88%. These recovery values were within the acceptable range of 70-120% as stipulated by Ribani et al. (2004) and INMETRO (2020). These promising results demonstrate that the method developed is highly reliable for the analysis of MT in biofloc water, showing both excellent precision and accuracy.

**Table 3:** Intra-day and inter-day precision and accuracy results of methyltestosterone in biofloc water at four different levels ($\bar{x}$±SD).

Methyltestosterone	Intra-day (*n*= 6)		Inter-day (*n*= 6)	
Spiked concentration (µg⋅L^−1^)	Measured concentration (µg⋅L^−1^)	Precision (RSD, %)	Measured concentration (µg⋅L^−1^)	Precision (RSD, %)
50	35.67 ± 2.97	8.32	35.39 ± 3.12	8.81
100	101.07 ± 7.60	7.52	99.88 ± 6.99	6.99
200	152.9 ± 11.96	7.82	153.6 ± 9.65	6.28

### Calibration curves, LOQ, and LOD

The analytical calibration curve for MT demonstrated excellent linearity. The linear regression equation was Y= 1267.3 + 112.82×C, where C is the concentration of MT in µg·L^−1^, resulting in a correlation coefficient (r) of 0.999 and a determination coefficient (R^2^) of 0.998. The curve was constructed using ten different concentration levels of MT (25, 50, 100, 200, 400, 600, 1000, 1500, 2000, 2500 µg·L^−1^), meeting the requirements set by the International Conference on Harmonization (ICH), the National Health Surveillance Agency (ANVISA), and the Group of Pesticide Residue Analysts (GPRA), which recommend a calibration curve with at least five concentration levels (ICH, 1994; ANVISA, 2003). This result complies with Brazilian regulations, which specify that the coefficient of determination (R^2^) should be between 0.90 and 0.99 (ANVISA, 2003). The limit of quantification (LOQ) of the calibration curve was determined to be 25 µg·L^−1^ based on a signal-to-noise ratio (S/N) of 10, while the limit of detection (LOD) was calculated at 10 µg·L^−1^, based on an S/N of 3. These results demonstrate that the method is suitable for quantifying MT in biofloc water.

### Matrix effect

In the matrix effect evaluation, the analytical curves of the solvent standards were compared with those of the matrix-matched standards. No significant statistical differences were found between the two sets of curves. As a result, the method was validated using calibration curves constructed in the solvent, as this approach yielded comparable results to those obtained in the matrix.

### Determination of MT in biofloc water samples

None of the biofloc water samples showed detectable residues of MT at 2 hours after the last feeding with hormone-containing food, so the samples collected after that were not analysed.

## Discussion

The hormonal treatment with MT in masculinization of Nile tilapia is used in fish farms spread in the whole world to obtaining uniformity in harvesting, higher growth rates, and avoiding overpopulation (Singh, 2013; Mlalila et al., 2015; Baroiller and D'Cotta, 2018). However, this practice, generally carried out in tilapia hatcheries, is not environmentally friendly due to periodic water changes, which can contaminate natural water sources with MT.

Water quality variables during the experiment were within reference values ​​for the species. The temperature was within the proposed experimental values. Although there were variations in oxygen concentrations depending on temperature, these were minimal and unable to promote any type of change in the other variables. Higher temperatures promote increased consumption due to accelerated metabolism (Volkoff and Rønnestad, 2020). Because of greater feed consumption at 28°C when compared with 25°C, there is an increase in excretion and formation of settleable solids, total suspended solids, and nitrate as well. The subtle and expressive reduction in pH and alkalinity, respectively, and a low ammonia and nitrite and an increase in nitrate at 28°C temperature is an indicative of a well-functioning nitrification process.

The increases in nitrite concentrations due to the increase in MT were minimal, lower than the reference value for the species (Table 1), and unable to cause any type of damage to the growth performance of the fish. Despite being increased at higher temperature, the concentration of nitrate in the water was well below the limit for the species, causing no changes in growth or even survival (Table 2).

These results demonstrate the important role of the BFT system in maintaining water quality parameters. Even though there was a greater feed intake at a temperature of 28°C, it was still not enough to harm the environmental conditions. On the one hand, this is due to the proportional incorporation of nitrogen in the body of the fish (heavier fingerlings) at the highest temperature and the ability of the system to convert or incorporate nitrogenous compounds.

The masculinization process of Nile tilapia in BFT using zero water exchange has become an alternative to the traditional method in tilapia hatcheries, as it allows the reduction and complete elimination of MT residues in the water, as demonstrated by Ramírez et al. (2024). These authors tested MT concentrations (0, 30, 40, 50 and 60 mg of MT ⋅ Kg^-1^ of feed), lower than those commonly used in nursery ponds (60 mg of MT ⋅ Kg^-1^ of feed, Baroiller and D’Cotta, 2018), and concluded that the lowest concentration (30 mg of MT ⋅ Kg^-1^ of feed) is efficient to achieve masculinization rate above 99%, considerably high and satisfactory for production. These results signaled that tilapia masculinization can be carried out in a more environmentally responsible way, using a zero-water exchange BFT, but also indicated that even lower concentrations should be tested. Furthermore, one fact in particular caught attention in the study by Ramírez et al. (2024), and gave rise to a new question: try to understand if the temperature used, 28°C, have influenced the masculinization rates, because control treatment, whose post-larvae received hormone-free feed, presented a number of males notably higher than what would correspond to half of the animals, as expected. Therefore, for the present study two hypotheses were tested. Whether it is possible to achieve high masculinization rates by further reducing the MT concentration in the feed and whether a higher temperature could contribute to this increase in a masculinization protocol of Nile tilapia larvae in a zero-water exchange BFT. To address these hypotheses, MT concentrations of 0, 10, 20, 30 and 40 mg ⋅ Kg^-1^ of feed were evaluated using two different temperatures, 25°C and 28°C.

Contrary to what was expected, at the same MT concentration, the use of 28°C *per se* does not increase the masculinization rate when compared to animals at 25ºC. The explanation may lie in the fact that at 28° C the need for MT for masculinization falls short of what is provided due to rapid growth, and, although the calculation for feed supply considers the mass gain of the previous week, it may still be insufficient for accompany an accelerated metabolism due to the higher temperature. Other possibility could lie in the fact that the tested temperature was lower than “masculinizing” temperature (over 32°C with best results around 36°C) (Azaza et al., 2008; Baroiller and D'Cotta, 2018), therefore, not high enough to make difference in masculinization rate over the use of MT on diet, apparently. However, the significantly higher male proportions (p < 0.05) observed in the controls (>50%) suggest that this tilapia lineage may be more thermosensitive, even at 25°C, as described by Bezerra et al. (2025), or may produce a higher proportion of males due to higher frequencies of autosomal alleles related to male sex differentiation (Baroiller and D’Cotta, 2018). This characteristic could also influence the male proportions observed in the treatments at the lower temperature. The same study should be done with other Nile tilapia lineages to evaluate more precisely the effects of the system, the protocol and the lineage.

Growth performance did not vary because of increasing MT concentration in the feed, corroborating the results of Ramírez et al. (2024), who also found no differences even when using higher MT concentrations (50 and 60 mg of MT ⋅ Kg^-1^ of feed). However, according to literature, changes in growth performance may occur due to the use of MT in different contexts, and due to interference from factors such as the species, the concentration of MT used in the feed, and the time of hormonal treatment (Karsli, 2021). Factors such as feeding rate, as well as, the production system used, can also determine the anabolic condition of MT in fish; however, in the case of tilapia masculinization in BFT, this effect has not been observed (Costa e Silva et al., 2022; Ramírez et al., 2024).

Water temperature and its variations directly affect the regulation of the metabolic rate and physiology of fish, with direct impacts on growth performance (Little et al., 2020; Schulte, 2011). The increase in consumption and growth in fish subjected to higher temperatures, if they are within the optimal range for each species, is widely reported in studies (Moura et al., 2007; Oliveira and Val, 2017; Amanajás and Val, 2023). In this study, although the growth performance and survival variables were not affected by the hormone concentrations tested, differences were observed between specific growth rate, final body weight and length of larvae produced at temperature of 25°C and 28°C, with the greatest development at highest temperature. It is possible to observe that in treatments at 28° C the final body weight of tilapia fingerlings was on average 2.7 times higher than reared at 25°C. In the experiment carried out by Azaza et al. (2008), 20-day-old tilapia were subjected to 22, 26, 30 and 34 °C to evaluate their growth performance and the fishes exposed to extreme temperatures (22 and 34 °C) had worse food utilization, lower growth and average final weight than at 26°C and 30°C, corroborating our results, where the lower temperature presented lower consumption and lower development. In a second experiment, the same authors tested temperatures 19, 32, 34 and 36.5°C in larvae during the first 28 days of life and found the lowest survival rates (60% and 75%) at extreme temperatures (19°C and 36.5°C, respectively), and the highest survival rates (92% and 93%) at intermediate temperatures (32°C and 34°C, respectively), indicating that the further the temperatures move away from the which is considered optimal, the lower the growth and the higher the mortality. Although in this atual experiment the highest temperature is within the optimal range (27°-30°C) for growth in Nile tilapia cited by Nivelle et al. (2019), the lowest temperature is two degrees below this minimum limit, however, no variations in survival were noticed between the 25°C and 28°C temperatures.

With the aim of reducing the concentration of MT in the masculinization of Nile tilapia, Ramírez et al. (2024) obtained rates greater than 99% of males using 30 mg ∙ Kg^-1^ of feed, without causing changes in water quality or growth variables, which means a significant reduction in environmental impacts. The authors also suggested that lower concentrations could be sufficient to obtain good masculinization rates, just as higher temperatures could allow an even greater reduction in MT concentration in the masculinization process. In the present study, it was possible to further reduce this concentration and confirm the environmentally friendly nature of masculinization in BFT. In this experiment, using MT concentrations of up to one-sixth (10 mg ∙ Kg^-1^ of feed) of that commonly used in tilapia hatcheries, 60 mg ∙ Kg^-1^ of feed (Baroiller and D’Cotta, 2018), it was possible to achieve masculinization rates greater than 97% and 99% in waters at 28°C and 25°C, respectively. There were no differences in masculinization rates between different concentrations of MT within the temperature 25°C nor between masculinization at 25°C and 28°C. However, at 28°C, there were higher masculinization rates at the concentration 40 mg ∙ Kg^-1^ of feed (100%) than at concentrations 10 and 20 mg ∙ Kg^-1^ of feed (97 and 96.9%, respectively). This small difference can be considered insignificant for a production scenario. It may be related to a greater difficulty in predicting larval growth, which is much greater at a temperature of 28°C, based on their performance in the previous week. This difficulty may have led to a slight underestimation of the required feed quantity to be offered, implying in a smaller amount of MT available to the larvae of the two treatments with lower MT concentration in the diet (10 and 20 mg MT ∙ Kg^-1^ of feed).

In a study with tilapia masculinization in a recirculation system, Wang et al. (2022) obtained, at a temperature of 28°C, masculinization rates of approximately 95% and 100% at concentrations of 10 and 20 mg ∙ Kg^-1^ of feed, respectively, corroborating our results using the same MT concentrations, and opposing the ideia that in BFT fish could have lower masculinization rates than in clear water due to consumption of the system's own flocs and reduction of feed containing MT, presented by David-Ruales et al. (2019) and by Costa e Silva et al. (2022). Furthermore, at concentrations below 10 mg ∙ Kg^-1^ of feed, the temperature of 36°C plays a predominant role in masculinization (Wang et al., 2022), which contrasts with our work, in which we did not observe any difference between the control treatments (without MT) at 25°C and 28°C, confirming that 28°C is not a sufficient temperature to promote an increase in masculinization rates.

Despite this, during the 28 days the amount of MT that entered the system was lower at 25°C, as lower temperatures reduce metabolism, reduce consumption, delay growth, and consequently, reduce the supply of feed containing MT. Ramírez et al. (2024) using 30 mg of MT ∙ Kg^-1^ of feed added the equivalent of 93 µg ∙ L^-1^ of MT over 28 days of experiment in the masculinization of tilapia in BFT with zero water exchange. In the present experiment, using a concentration of 10 mg of MT ∙ Kg^-1^ of feed in the masculinization of Nile tilapia in BFT at 28°C, considering the amount of feed that entered the system, it was possible to verify a reduction from 103.7 µg ∙ L^-1^ of MT to 26.3 µg ∙ L^-1^ of MT when comparing the same procedure being carried out using a concentration of 40 mg of MT ∙ Kg^-1^ of feed, a reduction of almost 4 times. Furthermore, at 10 mg of MT ∙ Kg^-1^ of feed and 25 °C of temperature, there was a reduction from 26.3 µg ∙ L^-1^ to 9.1 µg ∙ L^-1^ of MT when compared to a 28 °C of temperature, an additional reduction of almost 3 times and an accumulative reduction of over 11 times. The reduction was over 10 times compared to Ramirez et al. (2024). On the other hand, fish masculinized at 25°C will take longer to reach commercial weight, which can generate more operational costs during this additional period of time, which in this experiment took two weeks, considering the fish reached the minimum weight of 1 g on average. Thus, although the tilapia masculinization in a zero-water exchange BFT at 25°C seems to be more sustainable from an environmental point of view, this may not be true from an economic one. Thus, the results do not suggest that masculinization should be done at 25°C, but that even if the temperature is at 25°C it is possible to obtain batches of masculinized tilapia with a large proportion of males in a zero-water exchange biofloc system, a common situation in colder times of the year under greenhouses (autumn and spring), expanding the period of the year that high quality masculinized fingerlings could be produced.

For both temperatures (25 °C and 28°C), MT residues in the biofloc × water mixture were not detected 2 hours after the last supply of feed with hormone. This result indicates that temperature did not affect the degradation of MT, in the range of temperatures studied, since a natural degradation of MT probably occurs. For this study, the increased sensitivity of the HPLC technique was possible due to the use of the SPE column for extraction, purification and extraction of the samples, with the quantification and detection limits of MT being 50 µg·L^−1^ and 10 µg·L^−1^, respectively, an improvement compared to the method used by Ramírez et al. (2024), in which the liquid-liquid technique was used and the quantification and detection limits of MT were 200 µg·L^−1^ and 50 µg·L^−1^, respectively. This 5-fold increase in detection sensitivity was important to ensure greater safety and reliability regarding the absence of MT under the conditions presented. All possible concentrations after the introduction of feed containing MT over 28 days, either at 28 °C or 25 °C, except for the concentration of 10 mg MT ∙ kg^-1^ at 25 °C, were higher than the detection limit used. This means that there was some degradation process that prevented the accumulation of MT in the water, biofloc and organic residues of the system (since there was no clarification and water exchange during the experiment period), and that this process apparently was not affected by the variation in water temperature, the reason it was not necessary to analize the other biofloc × water mixture samples of posterior collection in time.

The absence of MT at the end of the masculinization period of Nile tilapia in the BFT system is probably due to a set of factors, which may be due to the transformation into metabolites after ingestion and elimination by the fish, or due to environmental processes. The degradation of MT resulting from photodegradation (Shore and Shemesh, 2003; Biswas et al., 2013) is unlikely to have occurred due to the difficulty of light penetration caused by the high turbidity characteristic of BFT, just as the adsorption of MT on sediment particles (Ong et al., 2012) should also not have occurred, since the water collected for MT analysis contained materials suspended by the action of bubbles caused by aeration and due to the protocol for MT extraction analysis, which considers this possibility. Therefore, it is assumed that the elimination of MT in water may occurred because of the activity of microorganisms capable of assimilating steroids (Green and Teichert-Coddington, 2000; Homklin et al., 2012) and degrading MT (Kolok and Sellin 2008; Srikwan et al., 2020). It is suggested that further studies be carried out to verify the feasibility of using aerobic bacterial strains as bioremediators in effluents containing MT. Thus, the inoculation of MT-degrading bacteria in water could be part of a tilapia masculinization protocol in BFT with zero water exchange, making this process more sustainable and MT contamination free in the environment.

The absence of MT in the water 2 hours after the end of the hormonal treatment period of Nile tilapia in the BFT system reinforces the possibility of the absence of environmental impacts caused by MT in masculinization, contrasting with what may occur when this practice is carried out in fish earthen ponds.

Although most likely all MT that entered the system was either biotransformed after being ingested by the fish or degraded by bacterial action, further studies are still important to verify whether there is no presence of MT at levels below the detection limit of 10 µg · L^−1^ after masculinization of tilapia in BFT. This confirmation is especially important because there are studies demonstrating that mollusks subjected to MT concentrations below 10 µg · L^−1^ had changes in reproduction (Czech et al., 2001; Janer et al., 2006; Rivero-Wendt et al., 2014). Mollusks can accumulate substances and facilitate the detection of minimal amounts of trace contaminants and are provided as good indicators for biomonitoring.

To better understand the processes under which MT is being eliminated, it would be interesting to conduct studies to identify and quantify the metabolites that are formed and to confirm the participation of bacterial activity in the degradation of MT during the masculinization of tilapia in BFT. Thereby, we come ever closer to producing an environmentally responsible masculinization model, since the use of MT is still widely used in tilapia production around the world, and, in most cases, it is carried out in earthen ponds, which generate effluents that may contain MT residues and cause environmental impacts. In a scenario of unstable international trade that may occur, developing a clean technology that overcomes possible trade barriers becomes increasingly necessary and important.

## Conclusion

The intra-laboratory validation of the analytical method for detecting and quantifying methyltestosterone via high-performance liquid chromatography with UV absorbance at 245 nm proved to be highly effective for analyzing residues in biofloc water.

This study demonstrated that it is possible to perform the masculinization procedure of Nile tilapia in the BFT system with zero water exchange with high male proportion (close to 100%), reducing the concentration of MT most used in production, 60 mg ∙ Kg^-1^ of feed, to one sixth, 10 mg ∙ Kg^-1^ of feed, both at 28 °C and even at a lower temperature of 25 °C, allowing the production of Nile tilapia masculinized batches of fingerlings during the period of the year within these temperatures. Adittionaly, at 25°C, the input of hormone in the system was lower and more environmentally friendly, however, the growth of fingerlings was also much lower, appearing that production in this condidtion may be less economically efficient than at 28°C. We also concluded that 2 hours after the last meal containing hormone there is no presence of MT residue in the water, which shows that the system itself, probably through bacteria in biofloc, can perform this function in both temperatures in a zero-water exchange situation.

## References

[B001] ANVISA (2003). Guia para validação de métodos analíticos e bioanalíticos..

[B002] Amanajás RD, Val AL (2023). Thermal biology of tambaqui (*Colossoma macropomum*): general insights for aquaculture in a changing world. Rev Aquacult.

[B003] APHA (1998). Standard methods for the examination of water and wastewater..

[B004] Avnimelech Y (2006). Bio-filters: the need for a new comprehensive approach. Aquacult Eng.

[B005] Avnimelech Y (2009). Biofloc technology: a practical guide book..

[B006] Azaza MS, Dhraïef MN, Kraïem MM (2008). Effects of water temperature on growth and sex ratio of juvenile Nile tilapia Oreochromis niloticus (Linnaeus) reared in geothermal waters in southern Tunisia. J Therm Biol.

[B007] Baroiller JF, D’Cotta H, Shved N, Berishvili G, Toguyeni A, Fostier A, Eppler E, Reinecke M (2014). Oestrogen and insulin‐like growth factors during the reproduction and growth of the tilapia Oreochromis niloticus and their interactions. Gen Comp Endocrinol.

[B008] Baroiller JF, D’Cotta H (2001). Environment and sex determination in farmed fish. Comp Biochem Physiol C Toxicol Pharmacol.

[B009] Baroiller JF, D’Cotta H, Wang HP, Piferrer F, Chen SL (2018). Sex control in aquaculture..

[B010] Bendschneider K, Robinson RJ (1952). A new spectrophotometric method for the determination of nitrite in sea water. J Mar Res.

[B011] Bezerra VM, Reis GPA, Melo CL, Menezes WF, Santos BD, Ferreira MP, Pires DC, Costa FFB, Ramírez JFP, Teixeira JP, Leal CAG, Ribeiro YM, Turra EM, Teixeira EA, Alvarenga ER (2025). A long-term high temperature on young Nile tilapia females affects its urogenital papilla morphology and future reproductive performance. Aquaculture.

[B012] Biswas S, Shapiro C, Kranz W, Mader T, Shelton D, Snow D, Bartelt-Hunt S, Tarkalson D, van Donk S, Zhang T, Ensley S (2013). Current knowledge on the environmental fate, potential impact, and management of growth-promoting steroids used in the US beef cattle industry. J Soil Water Conserv.

[B013] Borges AM, Moretti JOC, Mcmanus C, Mariante AS (2005). Produção de populações monosexo macho de tilápia-do-Nilo da linhagem Chitralada. Pesqui Agropecu Bras.

[B014] Cavatti A, Alvarenga ÉR, Toral FLB, Leite NR, Costa FFB, Goulart LQ, Correa RDS, Silva MA, Santos BD, Fernandes AFA, Turra EM (2023). Impact of selection for growth and stocking density on Nile tilapia production in the biofloc system. Aquaculture.

[B015] Conover WJ (1999). Practical nonparametric statistics.

[B016] Costa e Silva RZ, Alvarenga ER, Matta SV, Alves GFO, Manduca LG, Silva MA, Yoshinaga TT, Fernandes AFA, Turra EM (2022). Masculinization protocol for Nile tilapia (O. niloticus) in Biofloc technology using 17-α-methyltestosterone in the diet. Aquaculture.

[B017] Costa FFB, Alvarenga ÉR, Silva MA, Manduca LG, Leite NR, Bezerra VM, Moraes SGS, Goulart LQ, Menezes WF, Cavatti A, Campideli TS, Turra EM, Teixeira EA (2024). Soybean oil as diluent and vehicle for 17α-methyltestosterone in the masculinization of Nile tilapia (Oreochromis niloticus) in clear water and biofloc systems. Aquaculture.

[B018] Curtis LR, Diren FT, Hurley MD, Seim WK, Tubb RA (1991). Disposition and elimination of 17-α-methyltestosterone in Nile tilapia. Aquaculture.

[B019] Czech P, Weber K, Dietrich DR (2001). Effects of endocrine modulating substances on reproduction in the hermaphroditic snail Lymnaea stagnalis L. Aquat Toxicol.

[B020] David-Ruales CA, Betancur-Gonzalez EM, Valbuena-Villareal RD (2019). Sexual reversal with 17α-Methyltestosterone in Oreochromis sp.: comparison between recirculation aquaculture system (RAS) and Biofloc technology (BFT). J Agric Sci Technol A.

[B021] Di Rienzo JA, Casanoves F, Balzarini MG, Gonzalez L, Tablada M, Robledo CW (2015). InfoStat Version 2015.

[B022] Do Valle RCA, Silva MA, Alvarenga ÉR, Matta SV, Turra EM (2023). Water salinity during masculinization of Nile tilapia in biofloc system. Pesqui Agropecu Bras.

[B023] El-Sayed AFM (2019). Tilapia culture..

[B024] Farahmand H, Razak SHA, Hwang GL, Maclean N, Rahman MA (2007). Induction of tetraploidy in transgenic tilapia (Oreochromis niloticus) using physical shocks. Iran J Fish Sci.

[B025] Green BW, Teichert-Coddington DR (2000). Human food safety and environmental assessment of the use of 17α-Methyltestosterone to produce male tilapia in the United States. J World Aquacult Soc.

[B026] Guerrero RD, Shelton WL (1974). An aceto-carmine squash method for sexing juvenile fishes. Prog Fish-Cult.

[B027] Guiguen Y, Baroiller JF, Ricordel MJ, Iseki K, Mcmeel OM, Martin SA, Fostier A (1999). Involvement of estrogens in the process of sex differentiation in two fish species: the rainbow trout (Oncorhynchus mykiss) and a tilapia (Oreochromis niloticus). Mol Reprod Dev.

[B028] Hargreaves JA (2013). Biofloc production systems for aquaculture..

[B029] Homklin S, Ong SK, Limpiyakorn T (2012). Degradation of 17α- methyltestosterone by Rhodococcus sp. and Nocardioides sp. Isolated from a masculinizing pond of Nile tilapia fry. J Hazard Mater.

[B030] INMETRO (2020). Normalização e Qualidade Industrial, 2020. Orientação sobre validação de métodos analíticos..

[B031] ICH (1994). Validation of analytical procedures: text and methodology..

[B032] Islam MJ, Slater MJ, Bögner M, Zeytin S, Kunzmann A (2020). Extreme ambient temperature effects in European seabass, Dicentrarchus labrax: growth performance and hemato-biochemical parameters. Aquaculture.

[B033] Janer G, Lyssimachou A, Bachmann J, Oehlmann J, Schulte-Oehlmann U, Porte C (2006). Sexual dimorphism in esterified steroid levels in the gastropod Marisa cornuarietis: the effect of xenoandrogenic compounds. Steroids.

[B034] Karsli Z (2021). Effects of synthetic androgen (17α-methyltestosterone) and estrogen (17β-estradiol) on growth and skin coloration in emperor red cichlid, Aulonocara nyassae (Actinopterygii: Cichliformes: Cichlidae). Acta Ichthyol Piscat.

[B035] Khieokhajonkhet A, Sangphrom S, Aeksiri N, Tatsapong P, Wuthijaree K, Kaneko G (2022). Effects of long-term exposure to high temperature on growth performance, chemical composition, hematological and histological changes, and physiological responses in hybrid catfish ♂Clarias gariepinus (Burchell, 1822) ×♀C. macrocephalus (Günther, 1864). J Therm Biol.

[B036] Kolok AS, Sellin MK (2008). The environmental impact of growthpromoting compounds employed by the United States beef cattle industry: history, current knowledge, and future directions. Rev Environ Contam Toxicol.

[B037] Little AG, Loughland I, Seebacher F (2020). What do warming waters mean for fish physiology and fisheries?. J Fish Biol.

[B038] Lone KP, Ridha MT (1993). Sex reversal and growth of *Oreochromis spilurus* (Guenther) in brackish and seawater by feeding 17α-Methyltestosterone. Aquacult Fish Manage.

[B039] Mlalila N, Mahika C, Kalombo L, Swai H, Hilonga A (2015). Human food safety and environmental hazards associated with the use of methyltestosterone and other steroids in production of all-male tilapia. Environ Sci Pollut Res Int.

[B040] Monsees H, Klatt L, Kloas W, Wuertz S (2017). Chronic exposure to nitrate significantly reduces growth and affects the health status of juvenile Nile tilapia (Oreochromis niloticus L.) in recirculating aquaculture systems. Aquacult Res.

[B041] Monteiro MIC, Ferreira FN, Oliveira NMM, Avila AK (2003). Simplified version of the sodium salicylate method for analysis of nitrate in drinking waters. Anal Chim Acta.

[B042] Moura GS, Oliveira MGA, Lanna ETA, Maciel A, Maciel CMRR (2007). Desempenho e atividade de amilase em tilápias-do-nilo submetidas a diferentes temperaturas. Pesqui Agropecu Bras.

[B043] Nakamura M, Nozu R, Ijiri S, Kobayashi T, Hirai T, Yamaguchi Y, Seale A, Lerner DT, Grau GE (2015). Sexual characteristics of high-temperature sterilized male Mozambique tilapia, Oreochromis mossambicus. Zoological Lett.

[B044] Neuheimer AB, Thresher RE, Lyle JM, Semmens JM (2011). Tolerance limit for fish growth exceeded by warming waters. Nat Clim Chang.

[B045] Nivelle R, Gennotte V, Kalala EJK, Ngoc NB, Muller M, Melard C, Rougeot C (2019). Temperature preference of Nile tilapia (Oreochromis niloticus) juveniles induces spontaneous sex reversal. PLoS One.

[B046] Nozu R, Nakamura M (2020). Influence of prolonged cultivation on sexual characteristics of sterilized female tilapia, Oreochromis mossambicus, induced by high-temperature treatment. Aquaculture.

[B047] Oliveira AM, Val AL (2017). Effects of climate scenarios on the growth and physiology of the Amazonian fish tambaqui (Colossoma macropomum) (Characiformes: serrasalmidae). Hydrobiologia.

[B048] Ong SK, Chotisukarn P, Limpiyakorn T (2012). Sorption of 17α- Methyltestosterone onto soils and sediment. Water Air Soil Pollut.

[B049] Pandit NP, Bhandari RK, Kobayashi Y, Nakamura M (2015). High temperature induced sterility in the female Nile tilapia, Oreochromis niloticus. Gen Comp Endocrinol.

[B050] Piferrer F (2001). Endocrine sex control strategies for the feminization of teleost fish. Aquaculture.

[B051] Pörtner HO, Bock C, Mark FC (2017). Oxygen- & capacity-limited thermal tolerance: bridging ecology & physiology. J Exp Biol.

[B052] R Core Team (2021). R: A language and environment for statistical computing.

[B053] Ramírez JFP, Alvarenga ER, Costa FFB, Ferreira MP, Campos A, Pio NPB, Bezerra VM, Pires DP, Biscoto GL, Keller KM, Bezerra JF, Pelegrine DR, Salgueiro TM, Tadeu CMO, Turra EM (2024). Reduction of methyltestosterone concentration in feed during masculinization of Nile tilapia (Oreochromis niloticus) in biofloc system. Aquaculture.

[B054] Ribani M, Bottoli CBG, Collins CH, Jardim ICSF, Melo LFC (2004). Validação em métodos cromatográficos e eletroforéticos. Quim Nova.

[B055] Rivero-Wendt CLG, Borges AC, Oliveira-Filho EC, Miranda-Vilela A, Ferreira MF, Grisolia CK (2014). Effects of 17α-methyltestosterone on the reproduction of the freshwater snail Biomphalaria glabrata. Genet Mol Res.

[B056] Rothbard S, Zohar Y, Zmora N, Sivan BL, Moav B, Yaron Z (1990). Clearance of 17α-ethynyltestosterone from muscle of sexinversed tilapia hybrids treated for growth enhancement with two doses of the androgen. Aquaculture.

[B057] Rubalcaba JG, Verberk WCEP, Hendriks AJ, Saris B, Woods HA (2020). Oxygen limitation may affect the temperature and size dependence of metabolism in aquatic ectotherms. PNAS.

[B058] Sarker B, Das B, Chakraborty S, Hossain MA, Alam MMM, Mian S, Iqbal MM (2022). Optimization of 17α-methyltestosterone dose to produce quality mono-sex Nile tilapia (Oreochromis niloticus). Heliyon.

[B059] Schulte PM, Farrell AP (2011). Encyclopedia of fish physiology: from genome to environment..

[B060] Shore LS, Shemesh M (2003). Naturally produced steroid hormones and their release into the environment. Pure Appl Chem.

[B061] Singh AK (2013). Introduction of modern endocrine techniques for the production of monosex population of fishes. Gen Comp Endocrinol.

[B062] Srikwan P, Niamhom B, Yagi T, Thayanukul P (2020). Characterization of Methyltestosterone degrading bacteria isolated from tilapia masculinizing ponds: metabolic intermediate, glucose amendments effects, and other hormones transformation. Water Air Soil Pollut.

[B063] Thanasupsin SP, Chheang L, Math C (2021). Ecological risk of 17α-methyltestosterone contaminated water discharged from a full water recirculating earthen masculinization pond. Hum Ecol Risk Assess.

[B064] UNESCO (1983). Chemical methods for use in marine environmental monitoring..

[B065] Vidal LVO, Albinati RCB, Albinati ACL, Lira AD, Almeida TR, Santos GB (2008). Eugenol como anestésico para a tilápia-do-nilo. Pesqui Agropecu Bras.

[B066] Volkoff H, Rønnestad I (2020). Effects of temperature on feeding and digestive processes in fish. Temperature (Austin).

[B067] Wan ZY, Xia JH, Lin G, Wang L, Lin VCL, Yue GH (2016). Genome-wide methylation analysis identified sexually dimorphic methylated regions in hybrid tilapia. Sci Rep.

[B068] Wang JY, Ma YX, Hu QM, Peng F, Zhou M, Ji XS, Zhao Y (2022). All-male Nile tilapia larvae production using high-temperature and low dose of MTcombination treatment. Aquaculture.

[B069] Wedemeyer G (1996). Physiology of fish in intensive culture systems..

[B070] Yang S, Yang X, Li Y, Li D, Gong Q, Huang X, Wu J, Huang A, Kong F, Han X, Zeng X, Zhang C, Du J, Du X (2021). The multilevel responses of Acipenser baerii and its hybrids (A. baerii ♀× A. schrenckii ♂) to chronic heat stress. Aquaculture.

[B071] Yao ZL, Chen HJ, Zhao Y, Cao ZJ, Wang H, Ji XS (2021). A time course transcriptome analysis of brains from sex-undifferentiated Nile tilapia discloses genes associated with high-temperature-induced masculinization. Aquaculture.

[B072] Zhou Y, Zhang Y, Wei S, Li W, Li W, Wu Z, Jiang S, Lu Y, Xu Q, Chen L (2022). Reduced hypoxia tolerance and altered gill morphology at elevated temperatures may limit the survival of tilapia (GIFT, Oreochromis niloticus) under global warming. Fishes.

